# *De novo* transcriptome assembly using Illumina sequencing and development of EST-SSR markers in a monoecious herb *Sagittaria trifolia* Linn

**DOI:** 10.7717/peerj.14268

**Published:** 2022-10-26

**Authors:** Hanqing Tang, Josphat K. Saina, Zhi-Cheng Long, Jinming Chen, Can Dai

**Affiliations:** 1 School of Resources and Environmental Science, Hubei University, Wuhan, China; 2 Wuhan Botanical Garden, Chinese Academy of Sciences, Wuhan, China; 3 Hostgene Co., Ltd, Wuhan, China; 4 Hubei Key Laboratory of Regional Development and Environmental Response, Hubei University, Wuhan, China; 5 Current Affiliation: Centre for Integrative Conservation, Xishuangbanna Tropical Botanical Garden, Chinese Academy of Sciences, Menglun, China

**Keywords:** *Sagittaria trifolia*, EST-SSR markers, Transcriptome, Unigene

## Abstract

**Background:**

*Sagittaria trifolia* Linn. is a widespread macrophyte in Asia and southeast Europe and cultivated in parts of Asia. Although a few genomic studies have been conducted for *S. trifolia* var. *sinensis*, a crop breed, there is limited genomic information on the wild species of *S. trifolia*. Effective microsatellite markers are also lacking.

**Objective:**

To assemble transcriptome sequence and develop effective EST-SSR markers for *S. trifolia*.

**Methods:**

Here we developed microsatellite markers based on tri-, tetra-, penta-, and hexa-nucleotide repeat sequences by comparatively screening multiple transcriptome sequences of eleven individuals from ten natural populations of *S. trifolia*.

**Results:**

A total of 107,022 unigenes were *de novo* assembled, with a mean length of 730 bp and an N50 length of 1,378 bp. The main repeat types were mononucleotide, trinucleotide, and dinucleotide, accounting for 55.83%, 23.51%, and 17.56% of the total repeats, respectively. A total of 86 microsatellite loci were identified with repeats of tri-, tetra-, penta-, and hexa-nucleotide. For SSR verification, 28 polymorphic loci from 41 randomly picked markers were found to produce stable and polymorphic bands, with the number of alleles per locus ranging from 2 to 11 and a mean of 5.2. The range of polymorphic information content (PIC) of each SSR locus varied from 0.25 to 0.80, with an average of 0.58. The expected heterozygosity ranged from 0.29 to 0.82, whereas the observed heterozygosity ranged from 0.25 to 0.90.

**Conclusion:**

The assembled transcriptome and annotated unigenes of *S. trifolia* provide a basis for future studies on gene functions, pathways, and molecular mechanisms associated with this species and other related. The newly developed EST-SSR markers could be effective in examining population genetic structure, differentiation, and parentage analyses in ecological and evolutionary studies of *S. trifolia*.

## Introduction

The arrowhead (*Sagittaria trifolia* Linn.) is an emergent macrophyte in the Alismataceae family with a wide distribution across Asia and southeastern Europe (Chen, 1989). It is a common perennial herb, which is often found in freshwater wetlands, ditches, ponds, and rice paddies. The breeding system of *S. trifolia* is monoecious and reproduces both sexually by seeds and asexually via corms (Qin, Li & Dai, 2015). During anthesis, female flowers in an inflorescence open within one to three days followed by the blooming of male flowers which takes between four to ten days (Dai et al., 2018a). It is a self-compatible species pollinated by insects such as solitary bees, honeybees, syrphid flies, and other dipterans (Dai et al., 2018a). Achene fruits of *S. trifolia* ripen generally after three weeks of flowering. Some of its crop breeds have been planted as vegetables (corms), used in traditional Chinese herbal medicines, for water purification or ornamental purposes (Zheng et al., 2006; Li et al., 2009; Ahmed et al., 2019).

Previous research work on *S. trifolia* has devoted much effort to the antimicrobial activity and chemical composition (Yoshikawa et al., 1993; Yoshikawa et al., 1996; Zheng et al., 2006; Li et al., 2009), growth and reproductive success(Daimon, Miura & Tominaga, 2014), physiological and biochemical responses to diesel (Zhang et al., 2015), the internode elongation (Sasayama et al., 2016), resource allocation patterns within inflorescences (Dai et al., 2018a; Dai et al., 2018b), germination characteristics (Ozaki, Shimono & Tominaga, 2018), and biochemical, phytochemical, and mineral composition analyses (Ahmed et al., 2019). Despite its medicinal and economic importance, there is limited information regarding the molecular basis of this species. To date, in the case of *S. trifolia*, limited genomic data is available on public sources such as NCBI (National Center for Biotechnology Information), except for arbitrarily primed PCR (AP-PCR analysis), allozyme markers, inter-simple sequence repeat markers (ISSR), and chloroplast DNA (cpDNA *atpB*-*rbcL* intergenic spacers) region. These markers were used to examine phylogenetic relationship among *Sagittaria* species, clonal diversity and structure (Lyu & Wang, 2016), genetic variation (Chen, Gituru & Wang, 2007), and phylogeography among populations of *S. trifolia* (Chen et al., 2008).

Microsatellites also called simple sequence repeats (SSRs) are repetitive DNA elements with short sequence motifs of one to six base pairs (bp) scattered in whole genomes. They can be divided into expressed sequence tag (EST) and genomic SSR (Toth, Gaspari & Jurka, 2000). Compared with other different types of molecular markers such as restriction/amplified fragment length polymorphisms (R/AFLPs) and random amplification of polymorphic DNAs (RAPDs), SSRs are multiallelic in nature, relatively abundant, reproducible, with codominant inheritance and good genome coverage, making them fairly useful in genetic mapping, gene conservation, QTL analysis, as well as germplasm resources and pedigree analysis (Powell, Machray & Provan, 1996; Al-Atiyat, 2015). The next-generation sequencing (NGS), *e.g.*, RNA-seq (RNA sequencing), is a reliable, economical, and efficient approach to build transcriptomic data and detect SSRs for species devoid of genomic information (Wei et al., 2011; Chen et al., 2017). Such EST-SSRs have been developed in various plant species, for example, *Paeonia* species (He et al., 2020), *Sesamum indicum* (Wei et al., 2011), and *Brassica campestris* L. ssp. *chinensis* var. *utilis* (Chen et al., 2017). Due to the lower frequency of null alleles than traditional SSR, EST-SSRs have also been widely applied in parentage analysis to reduce errors and confusions (Ellis & Burke, 2007; Wen et al., 2013).

So far, the available codominant markers of *S. trifolia* include microsatellite markers developed by Wu et al. (2011) for *S. trifolia* var. *sinensis*, and EST-SSR markers for the same variant by You et al. (2020). These loci were applied to estimate the outcrossing rate of *S. trifolia* in natural populations (Li, Qin & Dai, 2015) and to estimate the seed outcrossing rate in distal and proximal fruits (Dai et al., 2018a). However, the available markers have been developed using a cultivated crop of *S. trifolia*, which may have undergone strong artificial selection and rapid evolution in the genome. Thus, progress in genetic diversity, natural selection, and fine-scale population structure in wild *S. trifolia* has been hampered by the lack of effective molecular markers. For instance, in estimating the outcrossing rate of *S. trifolia* (Dai et al., 2018a), only three SSR loci were successfully amplified and showed high polymorphism. Besides, the majority of available *S. trifolia* genomic SSRs have mono- and dinucleotide repeats (Wu et al., 2011), which usually yield less detectible difference in the size of alleles and thus allele miscalling (Diwan & Cregan, 1997; Song, Fickus & Cregan, 2002). Conversely, trinucleotide microsatellite loci have been reported to be more polymorphic, easily detected, and stable in various species (Cregan et al., 1999; Song, Fickus & Cregan, 2002; Li et al., 2020). Therefore, to aid studies on population genetics of *S. trifolia*, more SSR markers need to be developed, especially with longer repeat motifs, *i.e.*, tri-, tetra-, penta-, and hexanucleotide SSR loci. The current study reports on comparative transcriptome analyses of *S. trifolia* using RNA-seq tools, which highlights effective and highly informative SSR markers, thus providing the basis for future studies on mating and parentage patterns, genetic diversity, population differentiation, and phylogeography of the *Sagittaria* genus.

## Material and Methods

### DNA and RNA isolation

Young leaves of eleven *S. trifolia* individuals from ten natural populations in Hubei (population IDs are EZB, JZB, JZC, JZD, QCB, YXB, YXC, WHA, WHB, and WHD; see Zhou et al., 2020 for a map) were collected and frozen immediately in liquid nitrogen until RNA isolation. RNA extraction from the leaf sample was done with Total RNA Extraction Kit (Bio Teke, Beijing, China). The quality of total RNA was examined in 1.5% agarose gel electrophoresis, the concentration and purity were assessed using NanoDrop 2000 spectrophotometer (Thermo Fisher Scientific, Waltham, MA, USA). Finally, the RNA integrity was checked using an Agilent Bioanalyzer 2100 (Agilent Technologies) with an RNA Nano 6000 Assay Kit. Additionally, genomic DNA was extracted using a DNA extraction kit (MagicMag Genomic DNA Micro Kit) of Sangon Biotech (Shanghai, China).

### Transcriptome assembly and functional annotation

The methods for cDNA library construction for Illumina sequencing followed Saina et al. (2021). The library was sequenced by Illumina HiSeq 2500 sequencing platform and paired-end reads were generated. High-quality clean reads were produced from roughly 5GB raw data and the transcriptome assembly of *Sagittaria trifolia* was performed in Trinity program (Saina et al., 2021). For each gene, the longest transcript was selected as unigenes after assembly. To identify orthologous set of genes, the assembly of the first sample was used to map the reads of the other samples using Bowtie 2 software (Langmead & Salzberg, 2012). The unigene function was annotated using BLASTx (Altschul et al., 1997), with *e*-cut off value >10^−5^. All the gene sequences were then annotated using Clusters of Orthologous Groups and proteins (KOG); Kyoto Encyclopedia of Genes and Genomes (KEGG Ortholog database, http://www.genome.jp/kegg/), and Gene Ontology (GO). For GO annotations, TBtools v1.09 program was employed (Chen et al., 2020, https://github.com/CJ-Chen/TBtools.git).

### SSR loci identification, primer design, PCR amplification and validation of SSRs

The sequences of unigenes used for the identification of simple sequence repeats were scanned using microsatellite prediction server MISA (https://pgrc.ipk-gatersleben.de/misa). The EST-SSR primers were designed using Primer3 software (Untergasser et al., 2012, https://github.com/primer3-org/primer3/releases), with the following parameters: polymerase chain reaction (PCR) product length 100–300 bp, GC content 20–80%, annealing temperature 50−62 °C and primer size 18–26 bp. This procedure has screened out 86 microsatellite loci with long-repeat motifs (tri-, tetra- penta- and hexanucleotide; Table S2). For validation, considering the wide distribution of *S. trifolia*, 20 individuals were selected from seven natural populations located in central China (see the map in Zhou et al., 2020). We used genomic DNA to evaluate polymorphisms with 41 primers randomly selected from the 86 developed pairs.to. PCR reaction was conducted at a final volume of 25 µL reaction comprising 1 µL (50 ng) of genomic DNA, 0.2 µL Taq polymerase enzyme, 2.5 µL Taq buffer, 0.5 µL dNTPs, 1 µL of each primer, and 19.3 µL ddH_2_O. The PCR thermal profile for SSR primers was: initial denaturation step for 5 min at 94 °C; followed by 35 cycles of 94 °C for 40s, annealing for 45s at 60 °C, and extension at 72 °C for 40s; then a final extension step of 7 min at 72 °C. PCR reactions were carried out in a T100™ thermal cycler (Bio-Rad, Hercules, CA, USA). Two percent of agarose gel electrophoresis was used to verify that PCR has yielded successful amplification. Primers that generated clear and bright bands were selected and the forward sequence labeled with 6-FAM, ROX, HEX, or TAMRA fluorescent dyes at the 5′ end, and used for multiplexing. Amplified results were run on an ABI 3730 XL capillary electrophoresis analyzer (Applied Biosystems, Foster City, CA, USA) with GeneScan 500-LIZ size standard (Applied Biosystems, Foster City, CA, USA). Gene mapper 4.0 version (Applied Biosystems) was used to analyze the microsatellite marker profiles of all the 20 individuals.

### Data analysis

The parameters, including number of alleles (Na), effective number of alleles (Ne), observed (Ho) and expected (He) heterozygosities and Hardy-Weinberg equilibrium deviations were analyzed using GenAlEx 6.5 software (Peakall & Smouse, 2012, https://biology-assets.anu.edu.au/GenAlEx/Download.html). The Cervus 3.0 program (Kalinowski, Taper & Marshall, 2007; http://www.fieldgenetics.com) was used to estimate the polymorphic information content (PIC).

## Results and Discussion

### Transcriptome of *Sagittaria trifolia*

**Figure 1:**
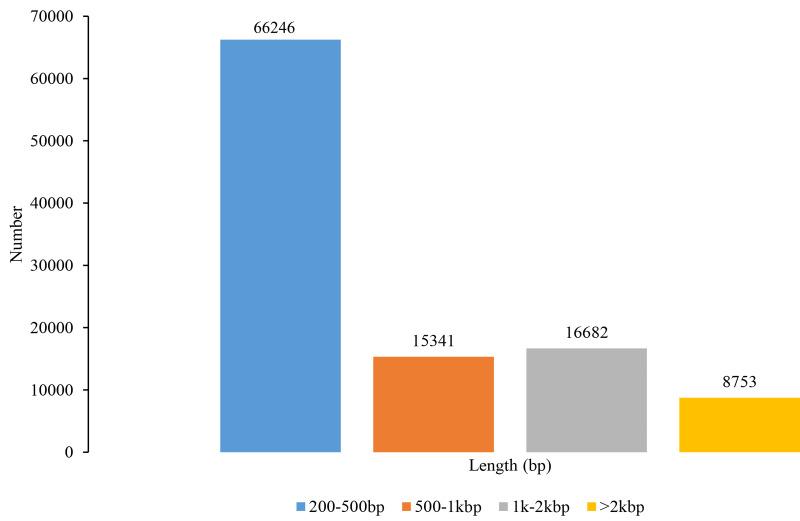
Length distribution of unigenes in *Sagittaria trifolia*.

In total, sequencing data generated 643,633,161 raw reads and 631,345,926 clean reads (Table S1). A total of 107,022 unigenes were *de novo* assembled, with a mean length of 730 bp, an N50 length of 1,378 bp, and a total length of 78,152,569 bp (Fig. 1). The Illumina paired-end reads as well as the assembly data can be found in the NCBI Short Read Archive (SRA) with the Bioproject accession PRJNA819828, SRA:  SRR18713852 – SRR18713862. The unigenes ranged from 201 bp to 12,680 bp and most of them had less than 1,000 bp. The N50 length within a range of 1,000 to 2,000 bp implies that the quality of transcriptome assembly is preferred and suitable for SSR marker development (He et al., 2020). Our reads quality and the number of unigenes have more than doubled those found in You et al. (2020) on *S. trifolia* var. *sinensis*. Therefore, the transcriptome assembly revealed by our study has the advantage of deeper data mining for genome evolution, gene structure, and function, as well as genetic marker development.

### Unigene annotations

**Figure 2:**
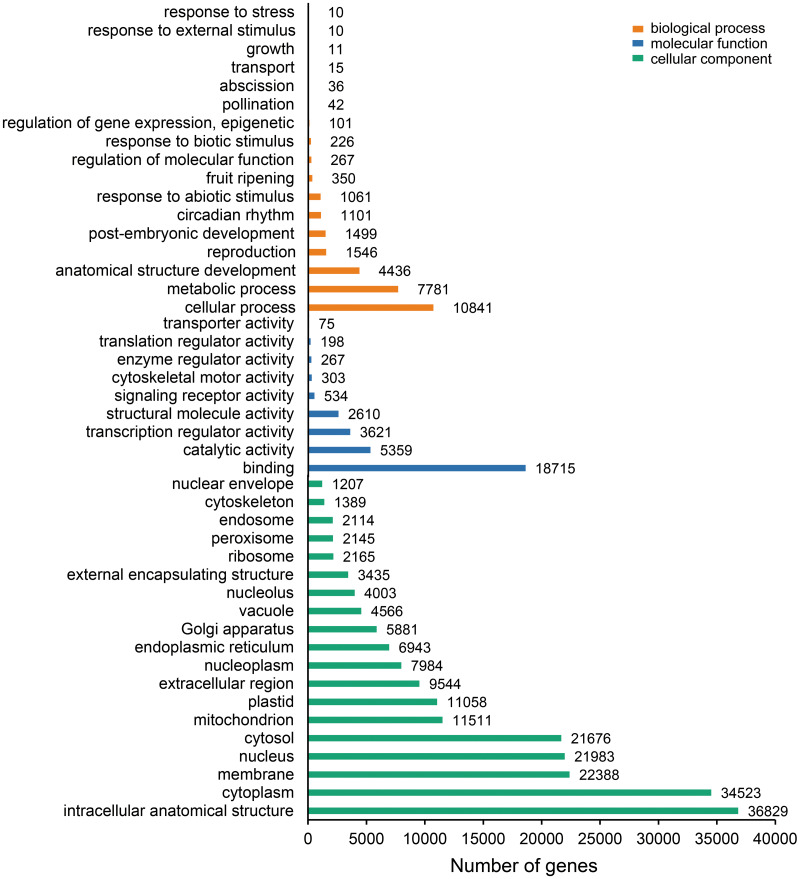
Gene Ontology (GO) function classification of the annotated unigenes in *Sagittaria trifolia*.

**Figure 3:**
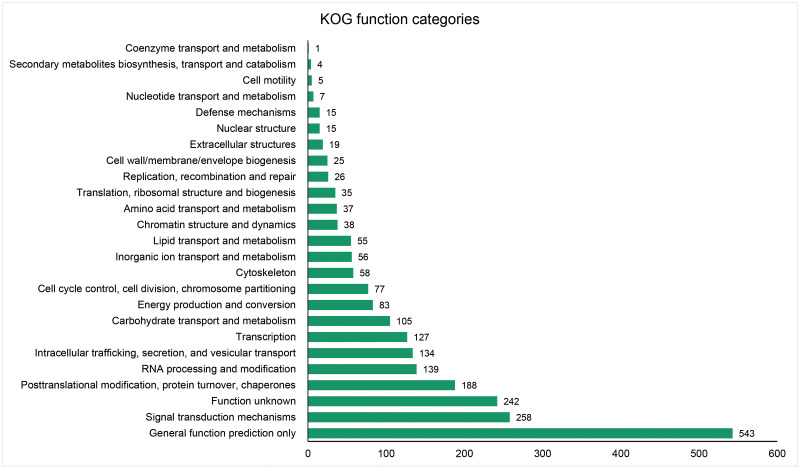
Distribution of the annotated unigenes of *Sagittaria trifolia* by KOG function.

The unigenes containing SSRs were classified using GO annotation to understand their potential functions (Fig. 2). Based on the broad distribution of unigenes among the three functional groups, 211,344 were classified into cellular component, accounting for 77.6% of all unigenes, followed by 31,682 (11.6%) in molecular function category and 29,333 (10.8%) in biological process. The biological process group had 17 subcategories. The highest number of genes were involved in cellular process (10,841) and metabolic process (7,781). For cellular component, which had 19 subcategories, intracellular anatomical structure (36,829) and cytoplasm (34,523) had the most gene numbers assigned, while the molecular function group (nine subcategories) had higher numbers of genes related to binding (18715) and catalytic activity (5,359) processes. Such distribution patterns have also been found in *Zantedeschia rehmannii* Engl. (Wei et al., 2016) and *Neottopteris nidus* (Jia et al., 2016). For KOG annotation (Fig. 3), a total of 2,292 contigs were identified and broadly divided into 24 function categories. The top five classes were: general function prediction only (543), signal transduction mechanisms (258), function unknown (242), posttranslational modification (188), RNA processing and modification (139). For KEGG (Table 1), a total of 3,575 contigs have been identified to participate in 138 pathways, which can be divided into five categories. Metabolism contained 10 subcategories, genetic information processing contained four subcategories, environmental information processing contained two subcategories, cellular processes contained one subcategory, organismal systems contained one subcategory. In metabolism pathway, about 24% contigs were assigned to carbohydrate metabolism, and other main processes involved amino acid metabolism and energy metabolism, together accounting for 53% of the contigs within this category. For genetic information processing pathway, translation and folding, sorting and degradation constituted 76% of all contigs. For environmental information processing pathway, the primary function took up 87% and was associated with signal transduction primary function resolved. These identified unigenes provide a basis for future studies to look into specific gene functions, pathways, and molecular mechanisms in *S. trifolia*. As a cultivated crop, its edible corms are mainly composed of starch. The synthesis pathways of carbohydrates are particularly worth investigating, which would benefit molecular breeding.

**Table 1:** KEGG Pathway classification of the annotated unigenes in *Sagittaria trifolia*.

Category	Subcategories	Number of pathways	Contig count
Metabolism	Carbohydrate metabolism	15	509
	Amino acid metabolism	14	326
	Energy metabolism	6	282
	Metabolism of cofactors and vitamins	11	238
	Lipid metabolism	13	222
	Metabolism of terpenoids and polyketides	12	131
	Biosynthesis of other secondary metabolites	18	129
	Metabolism of other amino acids	7	110
	Nucleotide metabolism	2	84
	Glycan biosynthesis and metabolism	9	76
Genetic Information Processing	Translation	5	432
	Folding, sorting and degradation	7	334
	Transcription	3	168
	Replication and repair	6	81
Environmental Information Processing	Signal transduction	3	148
	Membrane transport	1	23
Cellular Processes	Transport and catabolism	4	196
Organismal Systems	Environmental adaptation	2	86

### Distribution and characteristics of EST-SSR loci of *Sagittaria trifolia*

The predicted unigenes of *S. trifolia* yielded 21,756 SSRs in total, containing 12,146 mononucleotide, 3,820 dinucleotide, 5,114 trinucleotide, 455 tetranucleotide, 159 pentanucleotide, and 62 hexanucleotide repeats (Table 2). When mononucleotide repeats (55.83%) were excluded, the trinucleotide repeats account for the greatest portion (23.51%), followed by dinucleotide (17.56%) and tetranucleotide repeats (2.09%). The most dominant repeat motifs in the dinucleotide repeats were AG/CT (2,572, 67.33%) (Table 3), followed by AC/GT (720, 18.85%). Two most common trinucleotide motifs were AGG/CCT (1,074, 21%) and AGC/CTG (1,063, 20.79%). The most frequent repeats in the tetranucleotide motifs were AAAG/CTTT (88, 19.34%), and AGGG/CCCT (42, 9.23%) while the repeats of AATGG/ATTCC had the highest frequency (62, 38.75%) among all the pentanucleotide motifs (Table 3). The SSR repeat motif frequency shows quite species-specific patterns, probably due to the unique evolutionary history experienced by different plant genomes such as genome composition, structure or duplication events (Toth, Gaspari & Jurka, 2000; Varshney, Graner & Sorrells, 2005; He et al., 2020). Mono-, di-, and trinucleotide repeat motifs were found to be highly represented accounting for 96.90% in our study, which is similar to that of other plants such as *Paeonia* (94.53%), flowering Chinese cabbage (98.57%; Chen et al., 2017). Remarkably, among the dinucleotide repeat motifs, AG/CT was the most abundant motif and since CT and CTT microsatellite motif occurs frequently in the 5′UTRs (untranslated region) of a gene, it indicates that this repeat motif plays a crucial role in transcription and gene expression regulation (Wei et al., 2011).

**Table 2:** Type, number, and frequency of EST-SSRs of *Sagittaria trifolia*.

Repeats	Mono-	Di-	Tri-	Tetra-	Penta-	Hexa-	Total	Ratio (%)
5	NA	NA	2771	275	113	34	3193	14.68
6	NA	1098	1133	130	16	18	2395	11.01
7	NA	706	574	11	8	6	1305	6.00
8	NA	514	331	14	4	0	863	3.97
9	NA	304	63	11	7	0	385	1.77
10	2673	240	68	5	5	2	2993	13.76
11	1545	238	49	6	4	2	1844	8.48
12	955	249	28	1	1	0	1234	5.67
13	605	44	21	0	1	0	671	3.08
14	885	52	17	1	0	0	955	4.39
15	367	54	19	0	0	0	440	2.02
16	374	50	7	0	0	0	431	1.98
17	319	32	6	0	0	0	357	1.64
18	294	31	5	0	0	0	330	1.52
19	249	36	10	0	0	0	295	1.36
20	249	40	7	0	0	0	296	1.36
21	208	32	0	0	0	0	240	1.10
22	189	12	2	1	0	0	204	0.94
23	387	22	0	0	0	0	409	1.88
24	237	8	2	0	0	0	247	1.14
25	159	5	0	0	0	0	164	0.75
≥26	2451	53	1	0	0	0	2505	11.51
Total	12146	3820	5114	455	159	62	21756	
Ratio (%)	55.83	17.56	23.51	2.09	0.73	0.28		

**Table 3:** Distribution of EST-SSR motifs in the transcriptome of *Sagittaria trifolia*.

SSRs motif	Repeat number	SSRs motif	Repeat number	SSRs motif	Repeat number
A/T	10457	AAAC/GTTT	18	AGCG/CGCT	6
C/G	1689	AAAG/CTTT	88	AGGC/CCTG	15
AC/GT	720	AAAT/ATTT	23	AGGG/CCCT	42
AG/CT	2572	AACC/GGTT	6	ATCC/ATGG	36
AT/AT	476	AAGC/CTTG	5	CCCG/CGGG	7
CG/CG	52	AAGG/CCTT	35	AAAAC/GTTTT	9
AAC/GTT	281	AATC/ATTG	8	AAAAT/ATTTT	7
AAG/CTT	765	AATG/ATTC	8	AATGG/ATTCC	62
AAT/ATT	115	ACAG/CTGT	22	ACACC/GGTGT	5
ACC/GGT	566	ACAT/ATGT	33	AGAGG/CCTCT	10
ACG/CGT	243	ACCT/AGGT	5	AGGGC/CCCTG	5
ACT/AGT	62	ACGC/CGTG	10	AGGGG/CCCCT	11
AGC/CTG	1063	ACGG/CCGT	6	CCCGG/CCGGG	5
AGG/CCT	1074	ACTC/AGTG	8	AAGGAG/CCTTCT	6
ATC/ATG	396	AGAT/ATCT	29	ACCAGC/CTGGTG	5
CCG/CGG	549	AGCC/CTGG	26		

**Notes.**

SSR motif with repeat number less than 5 was not shown.

### Development and verification of *S. trifolia* EST-SSR primer pairs

During comparative screening on multiple transcriptome data, we particularly looked for SSR in *S. trifolia* with longer repeat motifs as they would result in greater precision and resolution in fine-scale population studies. A total of 86 microsatellite loci were identified with repeats of tri-, tetra- penta- and hexanucleotide (see Table S2). The 86 markers will provide a broad range of primers and repeat motifs for future studies to choose from. Of the 41 randomly selected EST-SSR primer pairs, 36 pairs were successfully amplified, whereas 28 produced stable and polymorphic bands of the expected lengths among twenty *S. trifolia* samples (Table 4; see primer information in Table S2). This was an exciting result as we were only able to successfully amplify three loci out of 17 (developed by Wu et al., 2011) using the same set of samples (Dai et al., 2018a). The polymorphic amplification efficiency found in our study (68.3%) is also higher than that of *Allium cepa* (60%; Li et al., 2015) and *Zantedeschia rehmannii* Engl. (56.2%; Wei et al., 2016). Interestingly, all of the above-mentioned studies did not differentiate SSR with trinucleotide (or longer) repeats from dinucleotide, which is considered less stable and thus might result in amplification failure (Zhao, Prakash & He, 2012). Therefore, the EST-SSR primers developed here are probably more applicable to studies using *S. trifolia* from other populations not sampled in our study.

**Table 4:** The genetic parameters (per locus) of 28 polymorphic SSR loci of *Sagittaria trifolia*.

Locus name	Na	Ne	Ho	He	PIC	HWE
NKKSSR001	5	2.45	0.70	0.59	0.56	Ns
NKKSSR003	4	2.24	0.45	0.55	0.51	Ns
NKKSSR004	8	4.91	0.50	0.80	0.77	***
NKKSSR007	2	1.41	0.25	0.29	0.25	Ns
NKKSSR009	6	2.63	0.60	0.62	0.59	Ns
NKKSSR010	4	3.39	0.45	0.71	0.65	*
NKKSSR011	5	3.94	0.35	0.75	0.70	***
NKKSSR013	6	3.31	0.30	0.70	0.66	**
NKKSSR014	5	3.38	0.65	0.70	0.65	*
NKKSSR017	6	3.36	0.40	0.70	0.65	**
NKKSSR018	4	1.87	0.60	0.46	0.42	Ns
NKKSSR019	6	4.04	0.80	0.75	0.72	Ns
NKKSSR020	11	5.48	0.75	0.82	0.80	Ns
NKKSSR021	6	3.40	0.35	0.71	0.66	***
NKKSSR022	3	1.83	0.25	0.45	0.40	**
NKKSSR023	4	1.80	0.45	0.44	0.41	**
NKKSSR024	8	4.68	0.80	0.79	0.76	Ns
NKKSSR025	8	4.79	0.90	0.79	0.77	Ns
NKKSSR026	5	3.21	0.70	0.69	0.65	Ns
NKKSSR027	10	4.06	0.85	0.75	0.72	***
NKKSSR028	4	2.85	0.45	0.65	0.59	Ns
NKKSSR030	3	2.12	0.47	0.53	0.47	Ns
NKKSSR032	4	3.14	0.70	0.68	0.63	Ns
NKKSSR036	5	3.56	0.50	0.72	0.67	Ns
NKKSSR038	3	2.06	0.75	0.51	0.44	Ns
NKKSSR039	5	3.52	0.70	0.72	0.67	Ns
NKKSSR040	3	1.56	0.25	0.36	0.31	Ns
NKKSSR041	2	1.41	0.25	0.29	0.25	Ns
Mean	5.2	3.10	0.54	0.63	0.58	

**Notes.**

Na observed number of alleles Ne expected number of alleles He expected heterozygosity Ho observed heterozygosity PIC polymorphism information content HWE Hardy-Weinberg equilibrium

Significant deviation from HWE at * *P* < 0.05, ** *P* < 0.01, *** *P* < 0.001, ns = not significant.

As for the genetic characteristics of sampled individuals, the observed (Ho) and expected (He) heterozygosity ranged from 0.25 to 0.90 and 0.29 to 0.82, with means of 0.54 and 0.63, both of which were higher than those reported for *Rhododendron rex* Lévl (Ho = 0.32, He = 0.37; Zhang et al., 2017) and *Elymus sibiricus* L.(Ho = 0.49, He = 0.59; https://www.frontiersin.org/articles/10.3389/fpls.2017.01664/full#B60). The number of alleles per locus ranged from 2 to 11 among 20 individuals, and the average number was 5.2, higher than those reported for *S. trifolia* var. *sinensis* (3.8 in Wu et al., 2011; 4.4 in You et al., 2020). This is probably because the variant studied previously is a cultivated crop in China, most of which are propagated using clones. According to the PIC value categorization (Botstein et al., 1980), a highly informative marker has a PIC value greater than 0.5, while a moderately informative marker has a PIC value ranging from 0.25 to 0.5, and a marker with PIC value lower than 0.25 is slightly informative. In this study, the PIC values of all loci were greater than 0.25, with a mean of 0.58 and was, again, higher than 0.22 in *S. trifolia* var. *sinensis* (Wu et al., 2011) and 0.42 in *Zantedeschia rehmannii* Engl. (Wei et al., 2016). All the resutls suggested that SSR with longer repeats developed by our study were highly informative markers. In addition, when we used the markers to conduct parentage analysis for *S trifolia*, loci with 3 bp repeats produced much lower mother-offspring mismatch rate than 2 bp (0.02 vs. 0.15; Dai et al., 2018a; H Tang, K Niu, P Zhou, C Dai, 2021, unpublished data). It highlights that the microsatellite markers developed here could be more effective and accurate in future genetic analysis of *S. trifolia*.

One caveat of the current study is that considering the broad distribution of *S. trifolia* across Asia and southeastern Europe, our sampling area is limited. This might produce a biased understanding of the characteristics of developed EST-SSR and an underestimation of the allele polymorphism. Nevertheless, transcriptome sequence is typically useful at species basis, especially as a reference for future studies. EST-SSRs are based on transcriptome other than genome sequence, which is more prone to neutral mutations (Lind & Gailing, 2013). Hence, the developed EST-SSR primers for *S. trifolia* should be more conserved and thus readily transferable to a wide range of populations.

## Conclusion

The present study developed SSR markers based on transcriptome sequencing data of *S. trifolia* and analyzed the distribution and characteristics of SSR loci. The specificity and polymorphism were verified in a subset of screened microsatellites. The results proved that transcriptome sequencing is an effective method for identifying molecular markers. This work lays a molecular foundation for studies on genetic diversity, ecology, and evolution of *S. trifolia*. Furthermore, the Alismataceae family, to which *S. trifolia* belongs, is placed at basal positions among Angiosperms. The transcriptomic data will provide an important molecular basis for studies on early angiosperm evolution, phylogeny, as well as aquatic adaptation.

##  Supplemental Information

10.7717/peerj.14268/supp-1 Supplemental Information 1 A summary of the Illumina sequencing quality *The first three-letter code denotes population sources, and the number after dash denotes genotype ID. See Zhou et al. (Zhou et al., 2020) for a map of populations.

10.7717/peerj.14268/supp-2 Supplemental Information 2 Characteristics of screened polymorphic EST-SSR loci in *Sagittaria trifolia* Note: The first 28 loci have been verified in this study. The remaining lack range sizes, which are marked as ‘NA’.

10.7717/peerj.14268/supp-3 Supplemental Information 3 Raw data of Table 3

## Additional Information and Declarations

### Data Availability

The following information was supplied regarding data availability: The data is available at NCBI BioProject: PRJNA819828.
