# Supplementary material for: De novo transcriptome assembly using Illumina sequencing and development of EST-SSR markers in a monoecious herb Sagittaria trifolia Linn
Source: PeerJ. 2022 Oct 26;10:e14268. doi: 10.7717/peerj.14268 (PMC9617548; doi:10.7717/peerj.14268)
Supplement: Supplemental Information 1 — *The first three-letter code denotes population sources, and the number after dash denotes genotype ID. See Zhou et al. (Zhou et al., 2020) for a map of populations. [file peerj-10-14268-s001.docx]

Supplementary Table S1 A summary of the Illumina sequencing quality.

| Sample name* | Total reads | Total mapped | Percentage |
| --- | --- | --- | --- |
| WHD-1 | 57,097,112 | 56,498,879 | 98.95% |
| WHB-2 | 54,595,562 | 53,584,754 | 98.15% |
| JZB-2 | 55,633,241 | 54,845,383 | 98.58% |
| JZB-3 | 46,565,662 | 45,327,687 | 97.34% |
| EZB-1 | 58,256,597 | 56,946,314 | 97.75% |
| QCB-1 | 71,547,777 | 70,329,847 | 98.30% |
| JZD-1 | 57,356,520 | 56,049,839 | 97.72% |
| WHA-1 | 53,541,785 | 52,480,420 | 98.02% |
| YXC-1 | 56,904,056 | 55,622,187 | 97.75% |
| JZC-1 | 57,356,520 | 56,049,839 | 97.72% |
| YXB1 | 74,778,329 | 73,610,777 | 98.44% |
| Summary | 64,363,3161 | 631,345,926 |  |

*The first three-letter code denotes population sources, and the number after dash denotes genotype ID. See Zhou et al. (Zhou et al. 2020) for a map of populations.
