# Supplementary material for: De novo transcriptome assembly using Illumina sequencing and development of EST-SSR markers in a monoecious herb Sagittaria trifolia Linn
Source: PeerJ. 2022 Oct 26;10:e14268. doi: 10.7717/peerj.14268 (PMC9617548; doi:10.7717/peerj.14268)
Supplement: Supplemental Information 2 — Note: The first 28 loci have been verified in this study. The remaining lack range sizes, which are marked as ‘NA’. [file peerj-10-14268-s002.docx]

Supplementary Table S2 Characteristics of screened polymorphic EST-SSR loci in *Sagittaria trifolia*

| Marker name | Forward primer | Reverse primer | Repeat motif | Tm (°C) | Size range (bp) | NCBI accession no. |
| --- | --- | --- | --- | --- | --- | --- |
| NKKSSR001 | CAGATCCGTCAAGCACAAGA | CGTGGTAGAAGAGGAGCAGG | CAG | 60 | 224-239 | ON088612 |
| NKKSSR003 | TCTGGTGCTTGTCGTCGTAG | AGGCAGATGGTAACAGGTGG | CCT | 60 | 250-267 | ON088613 |
| NKKSSR004 | AGAGGGTCGAGATCTGGGAT | TACATTGGTTTGCCCTGTCA | TTC | 60 | 291-324 | ON088614 |
| NKKSSR007 | CGGTACTGGTCGAGAAGAGC | TATCCTCCTTCCCATTTCCC | GGA | 60 | 218-224 | ON088615 |
| NKKSSR009 | TTTACTGGCCATCCGTTAGG | CCTATTCCTGCTCGATCTGC | GAG | 60 | 178-196 | ON088616 |
| NKKSSR010 | CGGAAGCCACAACTTAAAGG | CGTGGAAAAGATCACGGAAT | TCC | 60 | 214-223 | ON088617 |
| NKKSSR011 | CTTCTGCTCCTCCTCCTCCT | CGAATAGACGACAGGGTGGT | CTA | 60 | 251-263 | ON088618 |
| NKKSSR013 | GCTAGGTTGCTCCACTTTCG | CCTTCCTAACCGTTCAACCA | GAA | 60 | 192-211 | ON088619 |
| NKKSSR014 | TTCATCTGCACCAGCAAGAC | CAGCCGATCTGCATCAACTA | CGC | 60 | 238-250 | ON088620 |
| NKKSSR017 | CCACTCGCATCATCATCATC | GGTGTTGGCTGGTGAGATTT | CAT | 60 | 204-220 | OL901258 |
| NKKSSR018 | GCATGCTCTAGCCTTTACCG | GCGATGCCTCTTACTCGTTC | GTG | 60 | 256-277 | ON088621 |
| NKKSSR019 | AAAAGATGGTGTGGAGGTCG | GATCCTTTTCCGCTACCCTC | GCC | 60 | 206-237 | OL901259 |
| NKKSSR020 | TCGGTCGACGGTACACATAA | GTATGGGGGCGATTTAGGTT | GAAA | 60 | 279-305 | OL901261 |
| NKKSSR021 | AGAGACAAAGCCGAGACAGC | GAGGGTAGCAGAAGAATGCG | CTA | 60 | 259-285 | ON088622 |
| NKKSSR022 | CCTTTTTCTGCAGGTTGCTC | TTAGCTGCTGCTTCCCAAAT | GAA | 60 | 245-251 | ON088623 |
| NKKSSR023 | ATCATGCATCTAAGCGGACC | ATAAACTCGTTGGCGTGGAG | GCA | 60 | 173-178 | ON088624 |
| NKKSSR024 | GAGTGTGGCATCCGGTAGTT | CTGCTCTTATCCCTCAACGG | AAC | 60 | 253-298 | ON088625 |
| NKKSSR025 | GGCTATCCTGCTCTCCTCCT | ACCGCCTGCCATACAAATAG | CTC | 60 | 180-204 | OL901260 |
| NKKSSR026 | TGTTCTGGCAGAGTTTGACG | AGGTTGTTGCTGGGTTCATC | GCA | 60 | 199-210 | ON088626 |
| NKKSSR027 | TTCCAGACACCAACTCCTCC | GCCCAACCAAATGAAGAAGA | TTC | 60 | 123-150 | ON088627 |
| NKKSSR028 | ATCAGGAGGAGGACCAGGAT | CACACAGAGAGTCGAACGGA | CTG | 60 | 207-221 | ON088628 |
| NKKSSR030 | TGGACGAAGCACTTGAGATG | GTATCAACGATCAGCCGGAG | CTG | 60 | 297-303 | ON088629 |
| NKKSSR032 | TCACGTTTCTACCCCGTCTC | TCCCAGGTTTTGTTTGCTTC | CCTT | 60 | 246-258 | ON088630 |
| NKKSSR036 | GTATTGTGACGGGCACACAG | ATCATATGCCGAGATCCCAC | GGT | 60 | 272-286 | ON088631 |
| NKKSSR038 | ATTCCACTCGGGTTGATTCC | AATCCGAATGTAATGGAATGG | CATTC | 60 | 107-117 | ON088632 |
| NKKSSR039 | AGCCCGCAGCTTTATGAGTA | GGTTCCGAGATGATTAGCCA | CTC | 60 | 341-378 | ON088633 |
| NKKSSR040 | ACAAGGTGGGATGAAACTCG | ATCCTCTTCCATGCCCTTCT | CTC | 60 | 124-134 | ON088634 |
| NKKSSR041 | GCCATGATAGCGAGTGATGA | AGGAGAAGGAGAAGGAGAAGGA | CTTCTC | 60 | 107-122 | ON088635 |
| NKKSSR02 | TAGACCAGAAAATGCCCCTG | CGGATGGATGGAATCTTGTT | GAT | 60 | NA | ON125175 |
| NKKSSR05 | CGACTATGGCCTTGAGGTGT | CCTGAAGTTCTGGAACGAGC | AAGGAG | 60 | NA | ON125178 |
| NKKSSR06 | TGTCTGTGGCTGTGGATGTT | ACCGCAGGTTCAGACACAAC | TGG | 60 | NA | ON125180 |
| NKKSSR08 | AAAAGAACGATCCATGTGGC | CACACCCTCGTTCTCCATTT | GAT | 60 | NA | ON125184 |
| NKKSSR012 | GAAGAGGAGGAGGAGGAGGA | AGTGCGTTGCAAAGGAAAGT | GAG | 60 | NA | ON125189 |
| NKKSSR015 | CGTGGACAACTCCCAACTTT | TGTAATGCGGCGAAATATGA | ATG | 60 | NA | ON125193 |
| NKKSSR016 | GAACCATTGGACCCCTTTTT | GGTTGGGTTCCAGAACAAGA | CCAT | 60 | NA | ON125194 |
| NKKSSR029 | GAAAATCCGGAAGATGACGA | CATCTCCTTCCCATAGCTGC | CAG | 60 | NA | ON125210 |
| NKKSSR031 | ATTCTCGAGGCGTTTCTCAA | TCAATTGTAGCTGCTCCACG | AAT | 60 | NA | ON125211 |
| NKKSSR033 | GAGGAAGAGCCCGAAGAAGT | CGGTGTTGATGATTGTCCTG | GAG | 60 | NA | ON125214 |
| NKKSSR034 | ACATCCTTCCGTCCACTCAC | TTGCCTGTTTCCTTCCAATC | CGG | 60 | NA | ON125216 |
| NKKSSR035 | CTCGGAAACAGATAGAGCCG | AGTGTTTATTCCTGCCACCG | GAG | 60 | NA | ON125218 |
| NKKSSR037 | AGTGTCCAGCTCCTGCATCT | ACCTGGGGTGAACACTTCTG | GCG | 60 | NA | ON125225 |
| NKKSSR042 | AAGAAGGATCCATGTGGCTG | CTGCGAGTCAGTGTGCTCTC | GAT | 60 | NA | ON125176 |
| NKKSSR043 | TCTGGTGCTTGTCGTCGTAG | AGGCAGATGGTAACAGGTGG | CCT | 60 | NA | ON125177 |
| NKKSSR044 | GACGCAGATAGGATCGGGTA | CCTGAAGTTCTGGAACGAGC | AGGAGA | 60 | NA | ON125179 |
| NKKSSR045 | ATCATTGATTTGCGGGCTAC | AGGCAAAGAATGATGGATCG | GTAT | 60 | NA | ON125181 |
| NKKSSR046 | CAGCTTCCGTTGAAGTAGGC | CCTTCCTCTTCCTCCCGTAG | GAG | 60 | NA | ON125182 |
| NKKSSR047 | CGCTTGTTTTGGAAGAAAGG | CTCGAGTGAAGAGAGACGGG | TTC | 60 | NA | ON125183 |
| NKKSSR048 | ATCCACTCAAATCTCCGTCG | TTTACTGGCCATCCGTTAGG | CCT | 60 | NA | ON125185 |
| NKKSSR049 | TCTTGTACCTCGCGCTACCT | AGCCAAGGTAGGTCGGTCTT | CACG | 60 | NA | ON125186 |
| NKKSSR050 | TGATTGATCTTTTGCCCTCC | GAAGAGAATCTGCCGTCGTC | CGA | 60 | NA | ON125187 |
| NKKSSR051 | AGCTGCCAACAGAGGGAGTA | AGACGAGAAAGTTGCCAGGA | GCA | 60 | NA | ON125188 |
| NKKSSR052 | CGAGGTCAAGAAGAAGGCAC | GAATTATAAGCACGCACGCA | GTGC | 60 | NA | ON125190 |
| NKKSSR053 | GAGTGGTGGCTTTGGTTTGT | ACGTGGGATCAACAGGAGTC | GGA | 60 | NA | ON125191 |
| NKKSSR054 | CCCGTAATGAAAGAAACCGA | GTACGAGAGGACGACGGTGT | CCT | 60 | NA | ON125192 |
| NKKSSR055 | GAACCTGAACCTCCTCTCCC | GAAGAGATGGAGTGCAAGGC | CTC | 60 | NA | ON125195 |
| NKKSSR056 | GTGAGGAGGCTCTTGACGAC | GAGAACAGCAAACCTCTGCC | TGT | 60 | NA | ON125196 |
| NKKSSR057 | GTGGCAGAGGGTGTGAAGAT | CCCATCTCAGCTTCGTTCTC | AGGG | 60 | NA | ON125197 |
| NKKSSR058 | GAACAGCCACTTCTTCCAGC | ACAATGAGTAAGCCCCGTTG | GCCGG | 60 | NA | ON125198 |
| NKKSSR059 | CCTCCCAGTTGGAGATTTGA | GAAGGGAGAGACGGCTTTTT | ATAC | 60 | NA | ON125199 |
| NKKSSR060 | CCGCCCTACGTCTACAAGTC | TTTGTATGGTGGTGGTGGTG | CCA | 60 | NA | ON125200 |
| NKKSSR061 | GTCCCTTGCGAGAGAAACAG | CTTCATCAATGCATCCCCTT | TTC | 60 | NA | ON125201 |
| NKKSSR062 | TACCTCACCCCGTCAACTTC | CTGGAAGGTGAGCTTCTTGG | CAC | 60 | NA | ON125202 |
| NKKSSR063 | CGTGCATATTGTTCGATTGG | ACCCCTGTGGTTGTGTTGTT | ATC | 60 | NA | ON125203 |
| NKKSSR064 | ATTATTGGTCTCCGGGGTTC | CCTTTTCTTCTGGAGTTGCG | GCA | 60 | NA | ON125204 |
| NKKSSR065 | GACCTTGCTACGGCAAGAAG | TAGCATTCCACCACACGGTA | GCTG | 60 | NA | ON125205 |
| NKKSSR066 | AACCGACTCGTTGTTTGTCC | GAAAGTTGCCACTGGAGAGC | TGC | 60 | NA | ON125206 |
| NKKSSR067 | ATGGTTCCGCTACTCCCTCT | CGGAAAACGTGGAATCAGTT | CCA | 60 | NA | ON125207 |
| NKKSSR068 | GTTGTGGTCCGTGATGTCAG | TAGAAACACCATCGCAACCA | GTG | 60 | NA | ON125208 |
| NKKSSR069 | GCCAGCGAAGAAGAAGAAGA | ACTGAGGAAGGGGAGTCGAT | GAA | 60 | NA | ON125209 |
| NKKSSR070 | AAGACGAGGGTGAGGGATTT | AGCTTCGTTTCAAACTCCGA | TGC | 60 | NA | ON125212 |
| NKKSSR071 | AGAAATAATCCCCCACACCC | AGGGAGATGGAAGGAGGAAA | CACAC | 60 | NA | ON125226 |
| NKKSSR072 | GATCCTCTTTGTCAGCCTGC | GTATCGCCACATGTTTCGTG | GCG | 60 | NA | ON125213 |
| NKKSSR073 | GAGAGAGAGAGAGGCGACGA | TACTGCAGGAGGAAGGAGGA | TCC | 60 | NA | ON125215 |
| NKKSSR074 | TTAACCCAGCCCACTACCTG | GATTTTGCACCTGAACCGAT | CTG | 60 | NA | ON125217 |
| NKKSSR075 | GACGGAGGTGAGCTACTTGG | CCACAACCTCCTCCTGTTGT | AGC | 60 | NA | ON125219 |
| NKKSSR076 | GGTTGTGACGATGACAGTGG | GAACAACAAGCGTCGTAGCA | TAG | 60 | NA | ON125220 |
| NKKSSR077 | ATCCAGCAGCGATATGAACC | CTCGCTGTGAAAGCGTAAGA | AAT | 60 | NA | ON125221 |
| NKKSSR078 | GGAGGAGTCGTAGGGGAGTC | GACAGGGAGTCGAGGTGTGT | GCG | 60 | NA | ON125222 |
| NKKSSR079 | AACACGAACCGAAACAAAGG | GTTACACGCCCTTTTGCATT | AGG | 60 | NA | ON125223 |
| NKKSSR080 | TGTTGTAGCAGGCATGGAAA | ACTTGTGCCATGGCCATTAT | TCT | 60 | NA | ON125224 |
| NKKSSR081 | AACCCGTACCGTTTTGACAG | CCACTTTCATCCTCTTGGGA | ATA | 60 | NA | ON125227 |
| NKKSSR082 | TTCTGATGATCGAGCTGTCG | CAGAGCGTCCACACTTGAAA | GGT | 60 | NA | ON125228 |
| NKKSSR083 | GTTTGCTGTCCTCTTCCTGC | GCAAGGAAAGCATTTACCCA | GGC | 60 | NA | ON125229 |
| NKKSSR084 | GCTTGGCCTCTCTTCTCCTT | GTTCATCAGAAACCTCGGGA | GAC | 60 | NA | ON125230 |
| NKKSSR085 | TATCGGCAGCTGAAGGATTT | TACCGTGGGGTATGAGGTGT | ACT | 60 | NA | ON125231 |
| NKKSSR086 | GGAGGAGGAGGAGGAGAAGA | GGAGACGACCACCAGAAAAA | GGA | 60 | NA | ON125232 |

Note: The first 28 loci have been verified in this study. The remaining lack range sizes, which are marked as ‘NA’.
